# Waldenstrom Macroglobulinemia: Clinical Presentation, Diagnosis, and Management in an Elderly Male

**DOI:** 10.7759/cureus.44127

**Published:** 2023-08-25

**Authors:** Shahman Shahab, Dimitar I Semerdzhiev, James Reeves, Amy Daniel, David E Martin, Suporn Sukpraprut-Braaten

**Affiliations:** 1 Medicine, Arkansas College of Osteopathic Medicine, Fort Smith, USA; 2 Medicine, Unity Health, Searcy, USA; 3 Rural Primary Care, Unity Health, Beebe, USA; 4 Medicine, Kansas City University, Kansas City, USA

**Keywords:** pancytopenia, bruton tyrosine kinase inhibitor, cxcr4, kappa-lambda ratio, bone marrow aspirate, hyperviscosity syndrome, monoclonal igm gammopathy, large b-cell lymphoma, waldenstroms macroglobulinemia, waldenstrom

## Abstract

Waldenstrom macroglobulinemia (WM) is a rare lymphoproliferative disease that can have an ambiguous clinical presentation. A key component of the pathophysiology of WM is bone marrow infiltration, which most commonly presents as anemia. Other symptoms of WM tend to be generalized and non-specific, which presents a diagnostic challenge. This was the case with our patient as well, when he presented to our outpatient clinic with non-specific symptoms. We present a 79-year-old male with longstanding pancytopenia, polyarthralgia, bilateral pedal edema, decreased appetite, and increased bleeding from wounds. The patient had a complete blood count (CBC) and complete metabolic panel (CMP) done, confirming present anemia, which prompted inpatient treatment and an oncology workup, confirming WM. The patient began a zanubrutinib monotherapy regimen, showing improvement in his pancytopenia, polyarthralgia, and overall symptoms.

## Introduction

Waldenstrom macroglobulinemia (WM) is a lymphoproliferative disorder that involves B-cell proliferation in the bone marrow as well as immunoglobulin gammopathy, particularly IgM in the serum [1]. The condition is extremely rare and presents only 2% of all hematologic malignancies, with around 1000 to 1500 cases in the U.S. annually, translating to two to three people in 1,000,000 [2]. Due to its rarity, WM has an extremely heterogeneous presentation, which makes its diagnosis and treatment challenging. This condition remains incurable and without a clear prognosis for the people who suffer from it [2]. Although clinical literature is limited, it appears that WM often presents in a patient as non-specific symptoms such as generalized fatigue, weight loss, fever, and most commonly, anemia, which can even turn into pancytopenia [3]. These abnormalities can be detected with a regular blood workup due to pathologic infiltration and displacement of the bone marrow, resulting in abnormal blood work [3]. 

Accurate diagnosis and treatment are important for improving the prognosis of patients suffering from WM. A study looking at 5784 patients diagnosed with WM during the span of two decades saw an overall survival rate between six and eight years [4]. The research study noted that positive prognostic factors were age below 50 and being of African-American descent [4]. Other studies report negative prognostic factors for survival, such as advanced age and disease comorbidities [5]. Even with an accurate diagnosis, the treatment of WM, including inpatient, outpatient, and pharmacy costs, is very expensive; estimated average prices run approximately $27,000 per patient per month in the general population [6]. The cost of treatment increases when going from the first line to the third line of treatment as well [6]. These high costs continue to be an economic burden to patients, which leads to suboptimal patient adherence and poorer prognostic outcomes.

## Case presentation

We present the case of a 79-year-old male who presented to his primary care physician’s office with complaints of joint pain in his elbows, right knee, and bilateral shoulder blade pain. He also reported bleeding from his tongue and increased bleeding from wounds when he scratches himself, stating that it has worsened. The patient had a medical history of benign and innocent cardiac murmurs, essential hypertension, gastroesophageal reflux disease, low back pain, and hyperlipidemia. His surgical history included a left hip replacement. He did not report having any fatigue or myalgias.

Upon assessment, the patient appeared to have pale skin, which is not his baseline. Bleeding from his tongue was noted. He was also found to have bilateral ankle edema. He was given a preliminary diagnosis of anemia, and a blood test and labs were ordered to investigate his apparent anemic state further. The labs included a complete blood count (CBC) with auto differential, prothrombin time/international normalized ratio (PT/INR), activated partial thromboplastin time (aPTT), vitamin B12, folate levels, iron, total iron binding capacity (TIBC), serum ferritin, serum erythropoietin, and a complete metabolic panel (CMP). An outpatient CBC blood draw showed pancytopenia, and a recommendation for a hematology consult was made for further investigation. A recommendation was given for a blood transfusion, given the patient’s hemoglobin of 5.1 g/dL and an RBC of 1.6 th/uL. 

The patient was then referred to the local medical center, where he was admitted and administered 3 units of packed red blood cells (pRBC). At this stage, the patient’s findings looked most concerning for a primary bone marrow process such as myelodysplastic syndrome or acute myelogenic leukemia. A peripheral blood smear was obtained before transfusion, which showed leukopenia with a relatively proportional white cell differential count. No atypical leukocytes or blasts were identified. The red cells appeared normochromic and normocytic, with some anisocytosis. The platelets appeared significantly reduced in number, with a rare giant platelet noted. An increase in blood viscosity was also noted. A bone marrow biopsy and ultrasound were ordered.

The bone marrow biopsy showed markedly hypercellular marrow effaced by low-grade B-cell lymphoma. The cMyc proto-oncogene was negative. Flow cytometry of the bone marrow aspirate showed a flow differential of 0.5% blats, 17.8% granulocytes, 76.4% lymphocytes, 3.1% monocytes, and 1.7% CD45-negative elements. The myeloid-gated cells showed normal maturation patterns. The lymphoid-gated cells showed an abnormal B-cell population with kappa light chain restriction expressing CD45, CD19, and CD20 and lacking expression of CD5, CD10, and CD23, excluding follicular, small lymphocytic, and mantle cell lymphoma. The karyotype was normal. The MALT1 18q21 fluorescence in situ hybridization (FISH) was negative. The API2/MALT1 t(11:18) was negative. The patient’s bone marrow biopsy tested positive for the CXCR4 mutation, which is present in 28% of patients with WM and is either absent or present only in 7% of patients with other B-cell lymphomas. The bone marrow aspirate also tested positive for the MYD88 mutation, which is the most frequent abnormality in diffuse large B-cell lymphoma-activated B-cell-like subtypes detected in 40% of cases [7]. The MYD88 mutation is detected in approximately 90% of WM cases. The patient’s clinical picture along with these bone marrow biopsy findings point clearly toward WM [8].

Treatment

There is no cure for WM; therefore, the goal of treatment is to improve the quality and duration of life. Zanubrutinib is a highly selective Burton’s tyrosine kinase inhibitor indicated for WM [9]. The main options for treatment of WM are alkylating agents and monoclonal antibodies [10]. The first line of treatment for WM is R-CHOP (cyclophosphamide, doxorubicin, prednisone, rituximab, and vincristine), which is a combination of drugs containing rituximab, cyclophosphamide, doxorubicin hydrochloride, vincristine sulfate, and prednisone [6]. The second-line treatment for WM remains ibrutinib [6]. However, newer therapies such as zanubrutinib (Brukina®) have entered the market with better reduction of symptoms. During the ASPEN trial, a very good partial response (VGPR) was observed in 28% of patients treated with zanubrutinib compared to 19% in patients treated with ibrutinib [10]. Overall, the goal of therapy was to reduce disease activity and prevent any new symptoms. In a 25-month trial of the drug, three different response categories were established [10]. These categories defined by the International Workshops on WM (IWWM) are complete response, VGPR, and partial response [10]. A complete response is defined as no detectable disease and no new symptoms of WM [11]. A VGPR is defined as a small amount of detectable disease and no new symptoms of WM [11]. Partial response is defined as a detectable disease with no new symptoms of WM [11]. 

**Figure 1:**
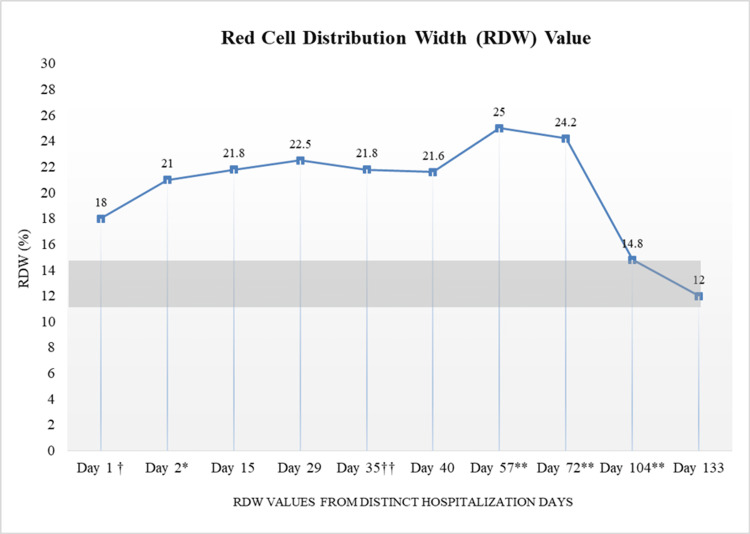
RDW values from distinct hospital visits Grey bar represents RDW normal range of 11.5% to 14.5% † First time patient presented to the clinic. †† Patient started on Zanubrutinib *Pre-transfusion hospitalization **Visits during which the patient received a blood transfusion RDW: Red cell distribution width

Our patient had a MYD88 and a CXCR4 mutation, which made him a good candidate for Burton’s tyrosine kinase inhibitors therapy [11]. He was started on zanubrutinib monotherapy. The daily dose was set at 80 mg once daily. In addition, the patient had also been receiving three transfusions in addition to treatment. He reported an improvement in his symptoms and a reduction in his pain levels. The patient also denied any adverse reactions or side effects from the treatment. There was an improvement in the patient's CBC panel (Table 1) and a reduction in the free kappa lambda ratio from 15.48 on day 57 to 9.08 on day 133. In addition, the patient's red cell distribution width (RDW) normalized post-initiation of treatment (Figure 1). His improvement in symptoms indicates that he is responding well to the treatment. He is currently in the intermediate risk group, and the five-year overall survival rate in his state is 78%, whereas the 10-year overall survival rate is 37%. He continues to follow up with his primary care physician as well as his oncologist.

**Table 1:** CBC from distinct hospital visits showing pancytopenia and trending CBC values † First time patient presented to the clinic. †† Patient started on Zanubrutinib *Pre-transfusion hospitalization **Visits during which the patient received a blood transfusion ***Lowest RDW showing efficacy of treatment HGB: Hemoglobin, MCV: Mean corpuscular volume, RDW: Red cell distribution width, PLT: Platelets, Lym: Lymphocytes, ANC: Absolute neutrophil count

Panel	CBC values from distinct hospital/clinic visits									
Hematology	Day 1 †	Day 2*	Day 15	Day 29	Day 35††	Day 40	Day 57**	Day 72**	Day 104**	Day 133
WBC th/uL (L = 4.8 H = 10.8)	3.2	3.1	2.9	2.8	3.3	5.3	4.4	5.4	5.3	5.2
RBC th/uL (L = 4.70 H = 6.10)	1.60	1.51	2.55	2.28	2.95	2.83	2.60	2.51	2.9	3.17
HGB G/dL (L = 13.5 H = 18.0)	5.1	4.8	7.4	6.8	8.5	8.4	8	8.4	10.2	11.1
MCV fl (L = 80 H = 94)	96.7	106	98	98	94	95	101	107	111	109
RDW % (L = 11.5 H = 14.5)	18.0	21.0	21.8	22.5	21.8	21.6	25.0	24.2	14.8	12***
PLT th/uL (L = 150 H = 450)	68	61	58	67	48	55	108	136	170	170
LYM% (L = 20.0 H = 40.0) %	42.2	44.9	41.8	39.0	42.2	52.2	49.7	40.8	35.0	36.6
ANC (L = 2.30 H = 8.10)	1.60	1.32	1.26	1.34	1.5	1.85	1.67	2.44	2.78	2.68

## Discussion

We diagnosed WM in our 79-year-old patient, which is a rare lymphoproliferative disease that can cause a varying degree of anemia due to bone marrow infiltration. Our patient had generalized symptoms, and his blood work showed pancytopenia, polyarthralgia, pedal edema, and increased bleeding from wounds. The patient’s clinical symptoms combined with initial laboratory findings prompted an oncology consultation, which confirmed WM. 

After the oncology referral, the patient was placed on zanubrutinib monotherapy, which improved his anemia as well as his joint pain. The patient also reported feeling less fatigued. The patient’s symptoms improved, and his lab results returned closer to baseline post-treatment with zanubrutinib. His RDW values returned to baseline as well. Patients who are treated with zanubrutinib can be placed into three general categories based on their responses to the treatment. Those categories are based on a 25-month-long clinical trial [10]. We cannot yet categorize our patient's response due to the length of treatment being shorter than 25 months at this time. However, the patient's response to the treatment as well as the symptomatic improvement observed are very promising. Since there is no cure for WM, the overall aim of treatment is to return to as close to baseline as possible, and that is our goal with our patient. Longer treatment with zanubrutinib has shown improved response and better patient outcomes, emphasizing the importance of early detection and treatment. Therefore, this patient is set to continue treatment with zanubrutinib. 

Due to the generalized symptoms of WM, it can provide a diagnostic challenge for healthcare professionals. Initial clinical presentation can often be mistaken for chronic anemia and may not be given the proper workup needed. This can delay proper diagnosis and treatment. Timely diagnosis and treatment of this condition can benefit patient outcomes, and early detection is important. Therefore, a standardized workup for this condition can benefit the early diagnosis and treatment of WM.

## Conclusions

Waldenstrom macroglobulinemia is an exceedingly rare condition that poses a set of challenges when it comes to correct diagnosis and treatment. Even with a correct diagnosis, the treatment of WM has a high financial burden, which decreases levels of therapy adherence in the general population. Once our patient was diagnosed with WM, he was placed on 80mg of zanubrutinib daily, which, unless covered by insurance, would be a cost-prohibitive treatment. 

We add this case report of WM to the literature due to the heterogeneity of patient presentation and the hope that it helps with early discovery and correct diagnosis. Our patient presented to the clinic with decreased appetite, joint pains, and increased bleeding time from superficial wounds, which have been worsening. During his clinical treatment, he had to be transfused twice with pRBCs in one month and had multiple hospital stays. These symptoms, multiple transfusions, and hospital stays prompted us to refer our patient to oncology, which sent him to obtain a bone marrow biopsy. The iliac crest biopsy displayed markedly hypercellular marrow effaced by a low-grade B-cell lymphoma. The pathology findings also reported an MYD88 mutation, an activating mutation found in roughly 93% to 97% of all WM cases. 

Symptoms such as anemia, pancytopenia, and increased bleeding, especially from the nose or gums, should raise concern for bone marrow pathologies. An adequate workup should be done, starting with a CBC panel and peripheral smear, followed by a bone marrow biopsy to confirm the diagnosis. Understanding the pathophysiology and clinical presentation of WM can help with an earlier, correct diagnosis, leading to a better long-term prognosis and quality of life.
